# Draft Genome Sequence of the Moderately Thermophilic Bacterium *Schleiferia thermophila* Strain Yellowstone (*Bacteroidetes*)

**DOI:** 10.1128/genomeA.00860-14

**Published:** 2014-08-28

**Authors:** Vera Thiel, Trinity L. Hamilton, Lynn P. Tomsho, Richard Burhans, Scott E. Gay, Robert F. Ramaley, Stephan C. Schuster, Laurey Steinke, Donald A. Bryant

**Affiliations:** a Department of Biochemistry and Molecular Biology, The Pennsylvania State University, University Park, Pennsylvania, USA; b Department of Geosciences, Penn State Astrobiology Research Center (PSARC), The Pennsylvania State University, University Park, Pennsylvania, USA; c Department of Biochemistry and Molecular Biology, University of Nebraska Medical Center, Omaha, Nebraska, USA; d Singapore Center for Environmental Life Sciences Engineering, Nanyang Technological University, Singapore, Singapore; e Department of Chemistry and Biochemistry, Montana State University, Bozeman, Montana, USA

## Abstract

The draft genome sequence of the moderately thermophilic bacterium *Schleiferia thermophila* strain Yellowstone (*Bacteroidetes*), isolated from Octopus Spring (Yellowstone National Park, WY, USA) was sequenced and comprises 2,617,694 bp in 35 contigs. The draft genome is predicted to encode 2,457 protein coding genes and 37 tRNA encoding genes and two rRNA operons.

## GENOME ANNOUNCEMENT

*Schleiferia thermophila* is a moderately thermophilic heterotrophic bacterium and currently is the only described species of the family *Schleiferiaceae* of the phylum *Bacteroidetes* (1). Strain Yellowstone was isolated from a phototrophic microbial mat in an effluent channel of Octopus Spring, an alkaline siliceous hot spring in the Lower Geyser Basin of Yellowstone National Park (44°32′2.833″ N, 110°47′53.352″ W, WY, USA).

Strain Yellowstone was purified from an enrichment culture using Percoll gradient centrifugation before DNA extraction. Identity and purity of the DNA sample was verified by 16S rRNA gene sequence analysis. Purified genomic DNA was subjected to bar-coded sequencing in an Illumina MiSeq instrument. The draft genome was assembled with Newbler (version 2.9, Roche) from 2,401,246 reads that had an average length of 301 bp. G+C content, read depth of scaffolds, and phylogenetic affiliation of BLAST hits were used to separate the genome sequence from contigs derived from contaminants resulting in 35 contigs comprising 2,617,694 bp.

Annotation using RAST (2) predicted 2,457 protein coding genes and 37 tRNA encoding genes. Based on coverage, two rRNA operons are predicted. Phyla-AMPHORA (3) identified 212 of 215 (98.6%) *Bacteroidetes*-specific phylogenetic marker genes. The remaining three marker genes were identified using blastP searches of the RAST annotated amino acid sequence output file. This analysis suggests that the draft genome is nearly complete. Based on gene content, strain Yellowstone is predicted to be an aerobic or microaerophilic heterotroph. Genes encoding glycolysis and gluconeogenesis pathways, tricarboxylic acid (TCA) cycle, and pentose phosphate pathway were complete. The genome lacks genes for nitrate reduction, suggesting this strain depends on reduced nitrogen sources. A complete respiratory electron-transport chain, including 6-subunit Na^+^-translocating NADH: quinone reductase (4, 5), succinate dehydrogenase, and two terminal oxidases are present. Similar to other genomes of members of the *Bacteriodetes*, strain Yellowstone apparently lacks ubiquinol:cytochrome *c* oxidoreductase but has an alternative complex III oxidoreductase. The genome lacks genes for ubiquinone biosynthesis but has a complete set of *men* genes for menaquinone biosynthesis. Menaquinone-6 was shown to be the major quinone in the *S. thermophila* type strain (1). The presence of a proteorhodopsin gene indicates that this organism may use light for establishment of an H^+^ gradient. The presence of anaplerotic enzymes (i.e., phosphoenolpyruvate [PEP] carboxylase and PEP carboxykinase), as well as carbonic anhydrase and a putative Na-dependent bicarbonate transporter, similar to other proteorhodopsin-containing members of the *Bacteroidetes*, suggests that light-stimulated anaplerotic inorganic carbon incorporation could occur (5).

Strain Yellowstone is bright orange in color and contains abundant carotenoids. High-performance liquid chromatography (HPLC) analysis suggested that most of the carotenoids have a myxol-like chromophore. Genes *crtBDEIYZA* and *cruF* for carotenoid biosynthesis were identified, which supports the assignment of myxol and its precursors as principal carotenoids (6). Precursors for carotenoids and other isoprenoid compounds are synthesized by the mevalonate pathway, which was complete except for mevalonate phosphate kinase (EC 2.7.4.2), which similarly has not been identified in other *Bacteroidetes* genomes (7).

### Nucleotide sequence accession numbers.

The draft genome sequence of *Schleiferia thermophila* strain Yellowstone has been deposited at DDBJ/EMBL/GenBank as a whole-genome shotgun project under the accession no. JDSI00000000. The version described in this paper is version JDSI01000000.
